# Effect of chronic high‐altitude hypoxia on retinal vascular phenotype, choroidal and retinal vasoreactivity in Peruvian highlanders

**DOI:** 10.1113/EP093967

**Published:** 2026-08-15

**Authors:** Aymeric Paillisser, Julien Vincent Brugniaux, Florence Berquet, Jeremy De Abreu, Stephen Hogg, Emanuele Trucco, Martial Geiser, Tom Macgillivray, Aurélien Pichon, Paul Robach, Benoit Champigneulle, Samuel Verges, Christophe Chiquet

**Affiliations:** ^1^ Department of Ophthalmology University Hospital of Grenoble Alpes, Grenoble Alpes University Grenoble France; ^2^ INSERM, U1300, CHU Grenoble Alpes, HP2 Laboratory Université Grenoble Alpes Grenoble France; ^3^ VAMPIRE Project, Computing, School of Science and Engineering University of Dundee Dundee UK; ^4^ Institute of System Engineering HES‐SO Valais Wallis Sion Switzerland; ^5^ VAMPIRE Project, Center for Clinical Brain Sciences University of Edinburgh Edinburgh UK; ^6^ Laboratoire MOVE, STAPS Université de Poitiers Poitiers France; ^7^ Site of the National School for Skiing and Mountaineering (ENSA) National School for Mountain Sports Chamonix France; ^8^ Department of Anesthesia and Critical Care CHU Grenoble Alpes Grenoble France

**Keywords:** high altitude, hypoxia, retinal vasculature

## Abstract

High‐altitude chronic hypoxia can induce excessive erythrocytosis (EE, defined as a haemoglobin concentration of ≥21 g/dL in men), leading to hyperviscosity and promoting endothelial dysfunction. We aimed to assess whether EE affects the retinal vascular phenotype and the ophthalmological vascular response to CO_2_. We conducted an ophthalmological cross‐sectional study among highlanders permanently living at 5100 m (La Rinconada, Peru). The central retinal artery equivalent (CRAE), central retinal vein equivalent (CRVE) and retinal vessel tortuosity were measured using semi‐automatic imaging software (VAMPIRE) from the diameters of the six largest arteries and veins on fundus images. Choroidal blood flow was assessed using laser Doppler flowmetry. Measurements were performed at rest and during a hypercapnic challenge (+10.1 ± 1.4 mmHg end‐tidal CO_2_)_._ Among the 62 included highlanders, 38 (61%) had EE. Compared with non‐EE, highlanders with EE exhibited higher CRVE (278 ± 25 vs. 249 ± 24 µm, *P *< 0.001), with no other significant ophthalmological differences. Resting CRVE was significantly correlated (all *P*‐values < 0.001) with haematocrit (*r *= 0.56), haemoglobin concentration (*r *= 0.65), blood volume (*r *= 0.55) and the arterial partial pressure of carbon dioxide (*r *= 0.46). Hypercapnia led to a moderate overall decrease in CRVE (−8.6 ± 22.0 µm, *P *= 0.02), without a specific effect of EE, and induced no other retinal vascular or choroidal blood flow changes in either group. We observed larger retinal vein diameters in EE highlanders compared with non‐EE. Although hypercapnia is known to increase retinal vessel diameter in healthy lowlanders, it selectively decreased CRVE in highlanders, irrespective of EE status. These findings suggest a retinal vascular dysfunction in highlanders, probably induced by chronic exposure to severe hypoxia.

## Abstract (highlights)

- **What is the central question of this study?**
  We investigated whether excessive erythrocytosis in highlanders leads to changes in ocular vasoreactivity within the retina and choroid.
- **What is the main finding and its importance?**
  At rest, highlanders with excessive erythrocytosis exhibited larger vein diameter, which was positively correlated with haemoglobin concentration, blood volume and arterial CO_2_ partial pressure. Highlanders, with or without excessive erythrocytosis, showed an absence of hypercapnia‐induced retinal vessel dilatation and even experienced a reduction in retinal vein diameter during CO_2_ inhalation. These data suggest that chronic exposure to hypoxia induces retinal vascular dysfunction in highlanders, irrespective of excessive erythrocytosis.

## 1 INTRODUCTION

Approximately 80 million people reside at high altitude (>2500 m) worldwide (Tremblay & Ainslie, 2021). As altitude increases, barometric pressure decreases and leads to a reduction in inspiratory oxygen partial pressure, arterial oxygen partial pressure (PaO_2_), arterial oxygen saturation and, therefore, oxygen delivery to the tissues. Consequently, highlanders permanently living in high‐altitude areas are chronically exposed to hypobaric hypoxia. Chronic mountain sickness (CMS) is a clinical syndrome affecting between 5% and 33% of high‐altitude residents (León‐Velarde et al., 2005), which is characterised by severe hypoxaemia, excessive erythrocytosis {EE, defined by consensus as a haemoglobin concentration ([Hb]) of ≥19 g/dL for women and ≥21 g/dL for men} and a range of clinical symptoms (Gatterer et al., 2024; León‐Velarde et al., 2005) that are assumed to be the consequence of EE and its subsequent increase in blood viscosity. The causal relationship between symptoms and EE in CMS has not been proved formally (Gatterer et al., 2024). EE and CMS have been shown to be associated with systemic vascular dysfunction (Savina et al., 2024) and/or pulmonary hypertension (Doutreleau et al., 2022) and may reduce life expectancy (Gatterer et al., 2024).

La Rinconada is considered to be the highest settlement in the world (5100–5300 m a.s.l.). Located in the south‐east of Peru, this gold‐mining city counts >50 000 inhabitants living in extreme hypobaric hypoxic conditions, with an inspiratory oxygen partial pressure of 50% compared with sea level (Champigneulle et al., 2024). The burdens of such a hypoxic environment include a broad increase in total haemoglobin mass and blood viscosity, even in healthy highlanders, leading to high levels of [Hb] and haematocrit (Champigneulle et al., 2024; Oberholzer et al., 2020). Consequently, both EE and CMS are highly prevalent in La Rinconada (Champigneulle et al., 2022; Hancco et al., 2020).

To the best of our knowledge, no data have been published before about choroidal vascularisation in high‐altitude dwellers considering their EE status. What we know is limited to the effect of acute hypoxic exposure, which leads to a significant vasodilatation of both retinal veins and arteries, in addition to an increase in vein tortuosity and in retinal blood flow (Brinchmann‐Hansen et al., 1989; De Abreu et al., 2025; Fallon et al., 1985; Louwies et al., 2016). We also recently reported a significant decrease in choroidal blood flow (ChBF; −13%) in lowlanders exposed for 10 days to high altitude (5100 m), measured using laser Doppler flowmetry (LDF; De Abreu et al., 2025).

Highlanders with EE or CMS are known to develop endothelial dysfunction (Rimoldi et al., 2012; Savina et al., 2024; Tremblay et al., 2019). We, therefore, hypothesised that EE highlanders would have dysfunctional vasoreactivity at the level of the retina and choroid, in comparison to non‐EE highlanders. Vasoreactivity of the retina and choroid blood vessels can be challenged easily using carbon dioxide (CO_2_), as shown by our group (Tonini et al., 2010) and others (Dorner et al., 2002; Venkataraman et al., 2008). Hypercapnia is known to increase the retinal arterial and venous diameters by 19%–25%, even at the level of the microvasculature (about +10% in precapillary arterioles and postcapillary venules; Dorner et al., 2002; Duan et al., 2017; Grunwald, 1993; Sponsel et al., 1992). Such vasodilatation is known to be triggered by different mechanisms involving interstitial acidosis (Pournaras et al., 2008), nitric oxide (Sato et al., 2003; Schmetterer et al., 1997), adenosine (Estevez & Phillis, 1997), ATP‐sensitive channels (Faraci et al., 1994) and prostanoids (Checchin et al., 2002; Pournaras et al., 1978). On the contrary, the vasoconstrictive effect of the orthosympathetic system projecting on the choroid might counterbalance the vasodilatory effects previously mentioned, preventing large changes in ChBF during hypercapnia (Gallice et al., 2017).

Thus, to test the hypothesis stated above, the aim of this experimental study was to define the retinal vascular phenotype (i.e., vessel diameter and tortuosity) and to investigate the CO_2_ vasoreactivity of retinal vessels and ChBF in highlanders with and without EE.

## 2 MATERIALS AND METHODS

### 2.1 Ethical approval

The study was conducted in accordance with the *Declaration of Helsinki* for research involving human subjects, except for preregistration in a database. Written informed consent was obtained from the participants after explanation of the study in their native language. The study protocol was approved by the local institutional review board (Universidad Peruana Cayetano Heredia, Lima, Peru; institutional review board number: 00003251).

### 2.2 Study population

Sixty‐two adult volunteers, permanently living in La Rinconada, Peru, at an altitude of 5100 m a.s.l., were included in the present cross‐sectional study, as part of a larger research programme (Expedition 5300) (Champigneulle et al., 2024). We included males, >18 years old, native Andeans from high‐altitude areas (i.e., born >3500 m a.s.l.), residing in La Rinconada for ≥3 years. Participants were recruited among the residents of La Rinconada who reported voluntarily to our temporary medical research laboratory. None of the participants had a medical history of cardiovascular, metabolic or respiratory diseases or a newly diagnosed or suspected medical condition at the time of clinical evaluation that could induce secondary polycythaemia or interfere with the diagnosis of EE and CMS. Exclusion criteria included heavy smoking (defined as smoking ≥20 cigarettes/day) and excessive alcohol consumption (defined as >21 drinks/week).

### 2.3 Study protocol

#### 2.3.1 Clinical examination

Demographic and clinical data (including age, body mass index and occupation) were recorded during an initial medical consultation. In particular, the seven clinical signs or symptoms (breathlessness and/or palpitations, sleep disturbance, cyanosis, venous dilatation, paraesthesia, headache and tinnitus) included in the CMS definition (León‐Velarde et al., 2005) were assessed and scored from zero (absence of symptom) to three (severe symptom). Three points were added to the resulting clinical score in the presence of EE ([Hb] ≥ 21 g/dL), thus obtaining the Qinghai CMS score. According to the diagnostic consensus (León‐Velarde et al., 2005), CMS diagnosis was retained in case of a Qinghai CMS score ≥ 6, including the presence of EE. The severity of CMS was defined as mild (CMS score = 6–10), moderate (CMS score = 11–14) or severe (CMS score ≥ 15). Arterial blood pressure and heart rate were measured after ≥5 min of sitting (A&D Company, Limited, Tokyo, Japan). Pulse oxygen saturation (SpO_2_) and end‐tidal CO_2_ partial pressure ($P_{\mathrm{ETC}O_{2}}$) were measured at baseline (IPM‐9800, Mindray, Shenzhen, China) and continuously during inhalation of the CO_2_ gas mixture (see below).

#### 2.3.2 Biological data

From a venous blood sample, haematocrit was measured in duplicate using the microcentrifuge method (Sigma 1–14, Sigma Laborzentrifugen, Osterode am Harz, Germany), and [Hb] was measured in duplicate using a co‐oximeter (ABL80, Radiometer, Copenhagen, Denmark). Arterial blood gases were obtained from a heparinised radial arterial blood sample analysed immediately with a portable blood gas analyser (i‐STAT, Abbott Point of Care, Princeton, NJ, USA) to measure arterial pH, PaO_2_ (in millimetres of mercury) and arterial partial pressure of carbon dioxide (PaCO_2_, in millimetres of mercury). Total blood volume was measured using an automated system (OpCO, Detalo Health, Copenhagen, Denmark) based on the carbon monoxide (CO) rebreathing method, as previously described (Oberholzer et al., 2020; Siebenmann et al., 2017).

#### 2.3.3 General ophthalmic examination

**FIGURE 1:**
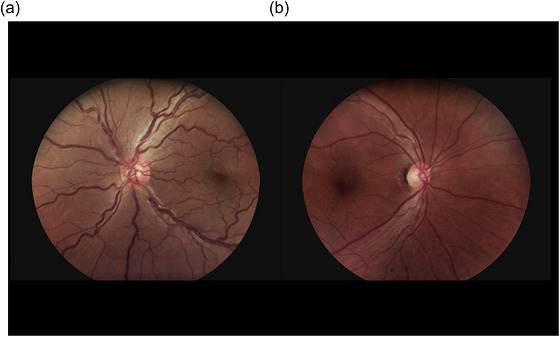
Example of a fundus image in highlanders with and without excessive erythrocytosis. (a) Left eye of a highlander with EE complicated by moderate CMS ([Hb] = 26 g/dL, haematocrit = 75%, CMS total score = 14) with CRAE = 211 µm, CRVE = 336 µm, TortA = 0.6 × 10^−4^ a.u. and TortV = 3.5 × 10^−4^ a.u. Note the significant increase in venous tortuosity. (b) Right eye of a healthy (without EE) highlander ([Hb] = 18 g/dL, haematocrit = 55%, CMS total score = 4) with CRAE = 169 µm, CRVE = 222 µm, TortA = 1.4 × 10^−4^ a.u. and TortV = 0.5 × 10^−4^ a.u. Abbreviations: a.u., arbitrary unit; CRAE, central retinal artery equivalent; CRVE, central retinal vein equivalent; EE, excessive erythrocytosis; TortA, arterial tortuosity; TortV, venous tortuosity.

The dominant eye of the participant (right eye for 49 subjects out of a total of 62) was determined by a pointing test at 5 m repeated four times. All ophthalmological examinations were afterwards conducted on the dominant eye. Each subject then underwent ocular examination that included refraction measurement (Retinomax K+ Screen, Right group, Tokyo, Japan), visual acuity [as a logarithm of the minimum angle of resolution (logMAR], intraocular pressure (Icare IC100 tonometer, Renevio Group, Helsinki, Finland), corneal thickness (Pocket II, Quantel Medical, Cournon d'Auvergne, France), axial length (Axis Nano, Quantel Medical, Cournon d'Auvergne, France), slit lamp examination before and after pupillary dilatation, and colour fundus imaging (45° Horus scope DEC200, Medimaging Integrated Solution Inc., Taiwan, resolution of 2560 × 1920 pixels; Figure 1).

#### 2.3.4 Retinal vascular phenotype assessment

Retinal vascular phenotype assessment was performed from fundus images that were centred on the optic nerve and secondarily analysed using the VAMPIRE software (v.3.2.0; McGrory et al., 2018). Baseline and hypercapnia testing images (cf. below) were analysed at the same time, single blinded to the experimenter, ensuring that the same sections of the vessels would be measured on both images. VAMPIRE v.3.2.0 measures morphological parameters of the retinal vessels in fundus images semi‐automatically. Details of VAMPIRE software have been reported elsewhere (McGrory et al., 2018; Mookiah et al., 2021). Briefly, the fundus images acquired by the portable camera were cropped to remove black borders, then resized to 2030 pixels × 1800 pixels resolution, preserving the aspect ratio if necessary. A small black cornice (15 pixels wide) was added on each side to avoid edge effects during image processing. Resizing is a standard operation by VAMPIRE software; it guarantees that all images (including those from different cameras where applicable) are processed on an equal footing, enabling consistent comparisons.

After locating the optic nerve and the centre of the macula, the software defines three circular zones around the optic disc (OD) as follows: zone A [annulus between 0.5 and 1.0 × OD diameter (ODD)], zone B (between 1.0 and 1.5 × ODD) and zone C (between 1.0 and 2.5 × ODD). Vessels are subsequently detected and labelled automatically as arterioles or venules. Measurements included CRAE and CRVE, averaging the diameters of the six largest arteries and venules, respectively (Knudtson's revised formulas; Knudtson et al., 2003), and arterial and venular vascular tortuosity. CRAE and CRVE were computed in zone B, and vascular tortuosity (TortA for arteries and TortV for venules) in zone C. Raw measurements of OD radius (ODr), OD to fovea distance (ODf), CRAE and CRVE were computed in pixels. Tortuosity is measured by an algorithm described by Lisowska et al. (2014) that reports a quantitative comparison of five algorithms for computing tortuosity. The best two methods, tortuosity density (Grisan et al., 2008; Lisowska et al., 2014) and slope chain coding (Bribiesca, 2013), are implemented and used in VAMPIRE software. The measurements used for this study are obtained from slope chain coding. Following the widely adopted formula by Knudtson et al. (2003), the conversion factor for the pixel‐to‐micrometre conversion was obtained by dividing the average ODD in pixels by the assumed average of the disc diameter in micrometres (1850 µm). The conversion factor was 6.28 µm/pixel.

#### 2.3.5 Choroidal blood flow measurement using LDF

The LDF used for subfoveal ChBF measurement has been described previously (Geiser et al., 1999; Riva et al., 2010). The technique is based on the Doppler effect of light (wavelength of 785 nm; power of 90 µW at the cornea) back‐reflected on moving erythrocytes. The light power value complies with the American National Standards Institute (ANSI) standard Z 136.1 for laser safety. The light scattered by the subfoveal choroid is focused onto an avalanche photodiode detector. The output photocurrent is sampled at a frequency of 240 kHz with a 16‐bit resolution and processed with Labview software (National Instruments, Austin, TX, USA) to determine the LDF parameters based on the theory of Bonner & Nossal (1990). These parameters, sampled at 17 Hz, are as follows: (1) velocity (ChVel, in hertz), a value proportional to the effective velocity of red blood cells; (2) volume [ChVol, in arbitrary units (a.u.)]; and (3) the relative blood flow (ChBF = ChVel × ChVol, in arbitrary units). The area of the probed surface was ∼280 µm in diameter for the choroid (Geiser et al., 1999). It is reasonable to assume that haematocrit remained constant during the experiment (Bonner & Nossal, 1990; Geiser et al., 2013; Riva et al., 2010). In this case, ChBF is proportional to the flow of RBCs. The LDF parameters were processed further to obtain mean values over the heart cycle. Briefly, ChBF was measured as follows. After pupil dilatation of the measured eye by instillation of one drop of tropicamide 0.5% (Mydriaticum; Thea, Clermont‐Ferrand. France), participants were asked to fix the probing beam, thus ensuring foveal location of the probed tissue volume (Chiquet et al., 2014). Entrance of the beam at the pupil was chosen to obtain a Doppler signal direct current output suitable for further processing. The intensity of the collected backscattered light via the direct current is a measurement of the optical properties of the local tissue under investigation. The software automatically rejects signals for which the direct current of the detected light is not within ±20% of its most frequent value; or LDF signal is absent; or the volume is suddenly too large, e.g., owing to micro‐saccades. The stability of the direct current value is a sign of good reproducibility. In our experience, the resolution of the measurement for LDF is ∼6%–10% in subjects measured at sea level (Mottet et al., 2018; Tonini et al., 2010).

The LDF instrument was mounted on a table equipped with a chin rest and was easily portable. Subjects were seated, and the operator aligned the instrument with the eye being tested. Furthermore, by adjusting the position of the instrument with respect to the eye, care was taken to keep the intensity of the backscattered light as constant as possible as assessed visually on the monitor of the LDF flow processor. Recordings of choroidal circulation for 90 s were obtained for each measurement.

#### 2.3.6 Vasoreactivity to CO_2_

During the CO_2_ vasoreactivity challenge, participants were asked to breathe through a face mask connected to a mixing chamber. The inspiratory side was connected to a room air–CO_2_ gas mixing chamber, allowing the inhalation of normocapnic and hypercapnic gas mixtures, and the expiratory side was used to determine $P_{\mathrm{ETC}O_{2}}$. Hypercapnia was achieved by increasing the inspiratory CO_2_ fraction to reach a $P_{\mathrm{ETC}O_{2}}$ target of +10 mmHg above the baseline value. Fundus images and 90 s LDF measurements were acquired after ≥5 min breathing room air through the mask (baseline) and in hypercapnic conditions (within a period of 2 min of stable hypercapnia).

### 2.4 Statistical analysis

Quantitative data were expressed as the mean ± SD. Baseline differences between EE and non‐EE highlanders were assessed using independent samples *t*‐tests with Welch's correction. Mean differences were reported with 95% confidence intervals (95% CI), and effect sizes were calculated using Cohen's *d*. Qualitative data were expressed as counts and percentages. Associations between vascular ophthalmic variables and biological parameters were assessed using Pearson's correlation coefficient or univariate linear regression. Changes in vascular ophthalmic variables between baseline and final measurements during the CO_2_ vasoreactivity challenge were compared between highlanders with and without EE using a mixed‐design ANOVA, with group (EE vs. non‐EE) as the between‐subjects factor and time (baseline vs. final) as the within‐subjects factor. Effect sizes were reported as partial eta squared (η^2^_p_) for main effects and interactions. In the case of a significant group × time interaction, *post hoc* comparisons were performed with Bonferroni correction.

Data analyses were conducted using R (v.4.2.2, The R Foundation for Statistical Computing, Vienna, Austria) and GraphPad Prism (v.10.6.1, GraphPad Software, Boston, MA, USA). All tests were two sided, and a *P*‐value of <0.05 was considered statistically significant.

## 3 RESULTS

### 3.1 General characteristics

Characteristics of the 62 highlanders included in the study are summarised in Table 1. Among them, 38 of 62 (61%) exhibited EE; almost all highlanders with EE (36/38, 95%) exhibited CMS. All participants were professionally active and involved in gold mining activities (75% miners, 25% technicians or security officers). Clinical and biological data are shown in Table 1. Highlanders with EE (*n* = 24, 39%) differed significantly from those without EE (*n* = 38, 61%) by a higher [Hb], haematocrit, PaCO_2_, diastolic arterial blood pressure and blood volume and by a lower PaO_2_ (Table 1).

**TABLE 1:** Clinical and biological characteristics of the 62 included highlanders and comparison between highlanders without (*n* = 24) and with (*n* = 38) excessive erythrocytosis.

Parameter	All participants (*n* = 62)	Non‐EE highlanders (*n* = 24)	EE highlanders (*n* = 38)	Non‐EE vs. EE comparison		
				*P*‐value	Cohen's *d*	Mean difference (95% CI)
Male sex, *n* (%)	62 (100)	24 (100)	38 (100)	_	_	_
Age, years	45 ± 11	42 ± 13	47 ± 9	0.184	−0.38	−4 (−10 to 2)
Height, cm	165 ± 6	166 ± 5	164 ± 6	0.109	0.41	2.3 (−0.5 to 5.1)
Weight, kg	72 ± 9	73 ± 10	72 ± 8	0.759	0.08	0.7 (−4.0 to 5.5)
BMI, kg/m^2^	26.6 ± 3.1	26.3 ± 2.7	26.8 ± 3.4	0.461	−0.18	−0.6 (−2.1 to 1.0)
Residency time in La Rinconada, years	14 ± 9	12 ± 8	15 ± 9	0.166	−0.36	−3 (−8 to 1)
Qinghai CMS score	8 ± 4	5 ± 3	10 ± 3	<0.001	−1.43	−4 (−6 to −3)
CMS status, *n* (%) No CMS Mild CMS Moderate‐to‐severe CMS	26 (42%) 24 (39%) 12 (19%)	24 (100%) _ _	2 (5%) 24 (63%) 12 (32%)	_	_	_
Systolic blood pressure, mmHg	120 ± 13	116 ± 11	123 ± 14	0.054	−0.50	−6 (−13 to 0)
Diastolic blood pressure, mmHg	87 ± 11	83 ± 9	89 ± 12	0.023	−0.58	−6 (−12 to −1)
Heart rate, beats/min	76 ± 12	74 ± 10	77 ± 13	0.259	−0.29	−3 (−9 to 3)
SpO_2_, %	81 ± 5	82 ± 4	81 ± 6	0.694	0.10	1 (−2 to 3)
Arterial pH	7.41 ± 0.04	7.42 ± 0.03	7.40 ±7.40 0.04	0.122	0.38	0.01 (0.00 to 0.03)
PaO_2_, mmHg	41 ± 5	43 ± 5	40 ± 5	0.019	0.68	3 (0.6 to 6)
PaCO_2_, mmHg	33 ± 4	32 ± 3	34 ± 3	0.003	−0.86	−3 (−5 to −1)
[Hb], g/dL	22.1 ± 2.4	19.6 ± 1.1	23.6 ± 1.6	<0.001	−2.82	−4 (−5 to −3)
Haematocrit, %	65 ± 8	57 ± 4	70 ± 6	<0.001	−2.36	−13 (−15 to −10)
Blood volume, mL	5867 ± 1208	5033 ± 713	6377 ± 1168	<0.001	−1.32	−1344 (−1839 to −849)
Blood volume, mL/kg^1^	82 ± 19	69 ± 7	90 ± 20	<0.001	−1.27	−21 (−29 to −14)

*Note*: Categorial data are reported as the absolute count and percentage. Continuous data are reported as the mean ± SD. Statistical comparisons were performed using independent *t*‐tests with Welch correction.

Abbreviations: BMI, body mass index; CMS, chronic mountain sickness; EE, excessive erythrocytosis; [Hb], haemoglobin concentration; PaCO_2_, arterial partial pressure of carbon dioxide; PaO_2_, arterial partial pressure of oxygen; SpO_2_, pulse oxygen saturation.

The two groups did not differ for visual acuity (0.9 ± 0.1 logMAR in non‐EE vs. 0.8 ± 0.2 logMAR in EE group; *P *= 0.081), spherical equivalent (−0.9 ± 1.4 dioptres in non‐EE vs. −0.8 ± 1.3 dioptres in EE group; *P *= 0.764), intraocular pressure (13.6 ± 3.1 mmHg in non‐EE *vs*. 13.6 ± 3.3 mmHg in EE group; *P *= 0.989), axial length (23.5 ± 0.6 mm in non‐EE vs. 23.2 ± 0.7 mm in EE group; *P *= 0.191) and corneal thickness (533.0 ± 32.1 µm in non‐EE vs. 540.6 ± 34.5 µm in EE group; *P *= 0.385).

### 3.2 Retinal vasculature phenotype

**FIGURE 2:**
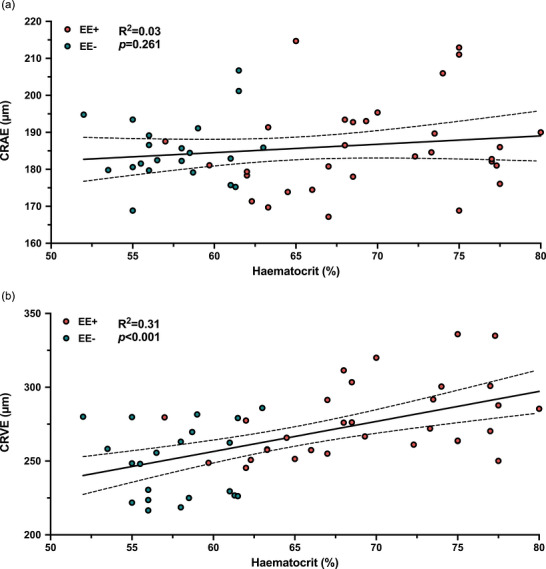
Association between haematocrit and retinal vessel diameters. Scatterplots illustrating the relationships between haematocrit and CRAE (a) or CRVE (b). Abbreviations: CRAE, central retinal arteriolar equivalent; CRVE, central retinal venular equivalent; EE, excessive erythrocytosis.

At baseline, non‐EE highlanders exhibited significantly lower CRVE than EE highlanders (Table 2); CRAE, TortA and TortV were not significantly different between the two groups. Positive correlations between baseline CRVE and haematocrit (*r *= 0.56; *P *< 0.001), [Hb] (*r *= 0.65; *P *< 0.001), blood volume (*r *= 0.55; *P *< 0.001) and PaCO_2_ (*r *= 0.46; *P *= 0.001) were observed in the overall population. No significant correlations (all *P*‐values >0.05) were observed between CRAE, TortA, TortV and [Hb], haematocrit or blood volume. Associations between haematocrit, CRAE and CRVE are shown in Figure 2.

**TABLE 2:** Baseline retinal vasculature phenotype in the 62 participating highlanders, and comparison between highlanders without (*n* = 24) and with (*n* = 38) excessive erythrocytosis.

Variables	All participants (*n* = 62)	Non‐EE highlanders (*n* = 24)	EE highlanders (*n* = 38)	Non‐EE vs. EE comparison		
				*P*‐value	Cohen's *d*	Mean difference (95% CI)
**Retinal vascular parameters**						
CRAE (µm)	186 ± 11	185 ± 9	186 ± 12	0.785	−0.07	−1 (−7 to 5)
CRVE (µm)	266 ± 28	249 ± 24	278 ± 25	<0.001	−1.16	−29 (−43 to −15)
TortA (a.u.)	2.0 × 10^−4^ ± 1.8 × 10^−4^	2.1 × 10^−4^ ± 2.3 × 10^−4^	2.0 × 10^−4^ ± 1.4 × 10^−4^	0.803	0.08	0.1 × 10^−4^ (−1.0 to 1.3 × 10^−4^)
TortV (a.u.)	1.1 × 10^−4^ ± 0.9 × 10^−4^	1.0 × 10^−4^ ± 0.6 × 10^−4^	1.1 × 10^−4^ ± 1.0 × 10^−4^	0.520	−0.16	−0.1 × 10^−4^ (−0.6 to 0.3 × 10^−4^)

*Note*: Data are reported as the mean ± SD. Statistical comparisons were performed using independent *t*‐tests with Welch's correction. CRAE and CRVE measurements were missing for 10 participants (3 without EE and 7 with EE), and tortuosity measurements were missing for 6 participants (2 without EE and 4 with EE) owing to insufficient quality of the captured fundus images.

Abbreviations: a.u., arbitrary unit; CRAE, central retinal artery equivalent; CRVE, central retinal vein equivalent; EE, excessive erythrocytosis; TortA, arterial tortuosity; TortV, venous tortuosity.

### 3.3 Effect of hypercapnia on retinal vasculature and choroidal blood flow

Insufficient quality of the fundus images during the hypercapnic challenge secondary to the difficulty of stabilizing the subject's head on the chinrest limited the analysis of CRAE/CRVE measurements to 43 paired values (69% of participants), the analysis of vessel tortuosity to 50 paired values (81% of participants) and the LDF measurements to 61 paired values (98% of participants).

During the hypercapnic test, in the overall population, $P_{\mathrm{ETC}O_{2}}$ increased by 10.1 ± 1.4 mmHg (95% CI: 9.7% to 10.5%; main time effect: η^2^_p_ = 0.981, *P *< 0.001), without any difference between EE and non‐EE highlanders (time × group interaction: η^2^_p_ = 0.001, *P *= 0.798). Overall, an absolute increase in SpO_2_ of 5.2% ± 5.0% (95% CI: 3.9% to 6.5%; main time effect: η^2^_p_ = 0.581, *P *< 0.001) was observed during the CO_2_ challenge. We assume that hypercapnia probably increased the respiratory rate of the subjects and enabled better oxygen gas exchange in the lungs, resulting in an increase in SpO_2_. The change in SpO_2_ was significantly different between groups (time × group interaction: η^2^_p_ = 0.131, *P *= 0.005), with EE participants showing a lower absolute increase in SpO_2_ during hypercapnia (+3.7%, 95% CI: 1.9% to 5.6%; *P *< 0.001) than non‐EE participants (+7.4%, 95% CI: 5.9% to 8.8%; *P *< 0.001).

**FIGURE 3:**
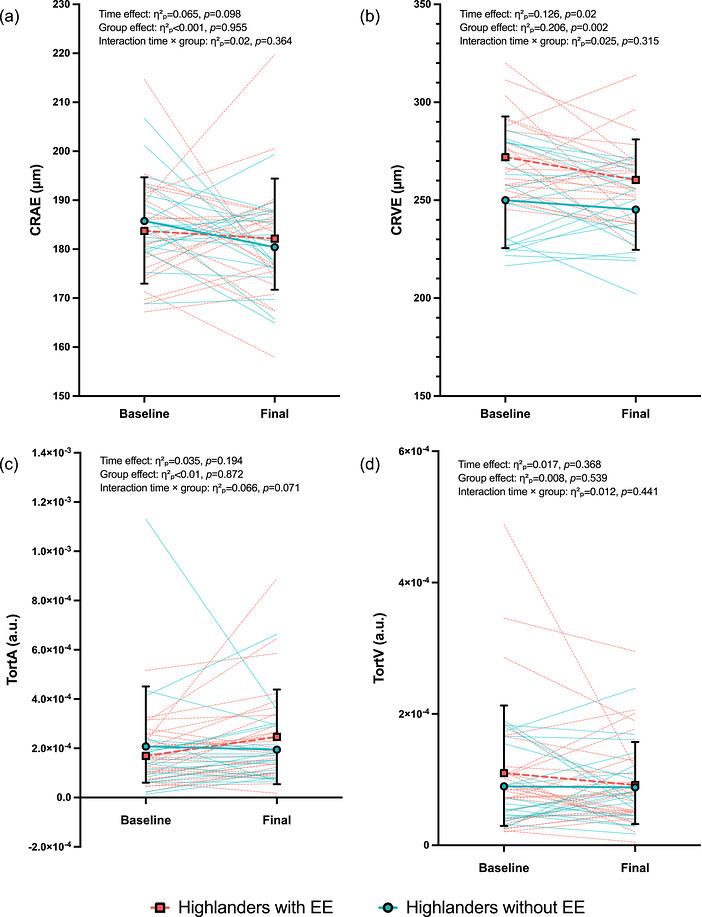
Change in retinal vasculature parameters in highlanders with and without EE during the CO_2_ reactivity challenge. Panels illustrate changes in CRAE (in micrometres; a), CRVE (in micrometres; b), TortA (in arbitrary units; c) and TortV (in arbitrary units; d) in highlanders with and without EE, measured at baseline and at the end of the CO_2_ reactivity test. In all panels, rectangles represent mean values for EE highlanders and circles for non‐EE highlanders, with error bars indicating the SD. Thick lines connect baseline and end‐test means (dashed for EE, continuous for non‐EE), and individual trajectories are shown as thin lines (dashed for EE, continuous for non‐EE). Changes in retinal parameters were analysed using a 2 × 2 mixed ANOVA. For each panel, *P*‐values and effect sizes (η^2^_p_) are reported for the main effects (time, group) and their interaction (time × group). Paired data were available for 43 of 62 highlanders (69%) for CRAE/CRVE measurements (24/38 EE and 19/24 non‐EE highlanders) and for 50 of 62 (81%) highlanders for tortuosity measurements (30/38 EE and 20/24 non‐EE highlanders). Abbreviations: a.u., arbitrary units; CRAE, central retinal arteriolar equivalent; CRVE, central retinal venular equivalent; EE, excessive erythrocytosis; TortA, arterial tortuosity; TortV, venous tortuosity.

**FIGURE 4:**
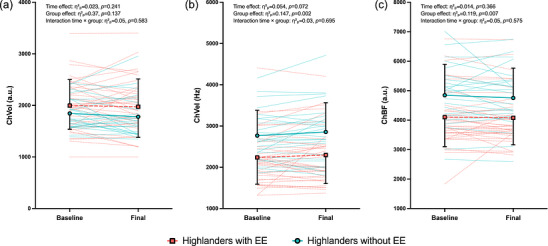
Change in choroidal blood flow parameters in highlanders with and without EE during the CO_2_ reactivity challenge. Panels illustrate changes in ChVol (inarbitrary units; a), ChVel (in hertz; b) and ChBF (in arbitrary units; c) in highlanders with and without EE, measured at baseline and at the end of the CO_2_ reactivity test. In all panels, rectangles represent mean values for EE highlanders and circles for non‐EE highlanders, with error bars indicating the SD. Thick lines connect baseline and end‐test means (dashed for EE, continuous for non‐EE), while individual trajectories are shown as fine lines (dashed for EE, continuous for non‐EE). Changes in choroidal parameters were analysed using a 2 × 2 mixed ANOVA. For each panel, *P*‐values and effect sizes (η^2^_p_) are reported for the main effects (time, group) and their interaction (time × group). Paired data were available for 61 of 62 highlanders (98%); one measurement was missing in one highlander with EE owing to a dense cataract. Abbreviations: a.u., arbitrary units; ChBF, choroidal blood flow; ChVel, choroidal velocity; ChVol, choroidal volume; EE, excessive erythrocytosis.

In the overall population, the hypercapnic test did not induce significant changes in CRAE (Figure 3a), vascular tortuosity (Figure 3c,d) or choroidal blood flow (Figure 4) parameters, but induced an overall moderate decrease in CRVE (−8.6 ± 22.0 µm, 95% CI: −15.4 to −1.8 µm; main time effect: η^2^_p_ = 0.126, *P *= 0.02), without any difference between groups according to the EE status (time × group interaction: η^2^_p_ = 0.025, *P *= 0.315).

## 4 DISCUSSION

In the highlanders examined, we demonstrated that: (1) retinal vein diameter was significantly correlated with haematocrit and blood volume; (2) EE was associated with a significant increase in vein diameters vs. non‐EE; (3) EE was not associated with any change in choroidal or retinal vasoreactivity to CO_2_; and (4) hypercapnia selectively decreased retinal vein diameter independently from EE status, suggesting an abnormal retinal vascular reactivity in permanent high‐altitude inhabitants.

Like others (Ma et al., 2023), we observed a higher CRVE in EE highlanders than in non‐EE. Peripheral venous dilatation has also been described in EE and CMS, and it might be attributable to the large increase in the total blood volume (Table 1; Villafuerte & Corante, 2016). Several underlying mechanisms can also be suggested, such as higher blood viscosity in EE subjects (Stauffer et al., 2020) and some degree of retinal vein compression in the optic nerve, as suggested by an increase in retinal nerve fibre layer following rapid ascent to high altitude under acute hypoxia (Tian et al., 2018).

We hypothesised that there would be a different vascular response to a vasodilatory stimulus depending on EE status because vasodilatation is an adaptive mechanism to hypoxia and EE highlanders probably had surpassed adaptive capacities at both the systemic and cerebral levels. Regarding the former, in comparison to healthy lowlanders, highlanders exhibit more systemic oxidative–nitrosative stress than normoxic controls; this seems even more pronounced in CMS subjects than in healthy highlanders (Bailey et al., 2013), owing to a decrease in vascular NO bioavailability and blunted cerebral perfusion and vasoreactivity to CO_2_ (Bailey et al., 2019). We did not find any difference between EE and non‐EE subjects for choroidal and retinal vasoreactivity to CO_2_, although a reduction in central CO_2_ sensibility, an increase of blood viscosity and systemic vascular dysfunction (impaired flow‐mediated dilatation, greater pulse wave velocity and greater carotid intima–media thickness) have previously been associated with CMS or EE (León‐Velarde & Richalet, 2006; Léon‐Velarde et al., 2003; Rimoldi et al., 2012; Savina et al., 2024; Tremblay et al., 2019). Consistent with our findings, Bailey et al. (2019) did not find any significant difference in vascular reactivity of the middle cerebral artery by transcranial Doppler measurements following a hypercapnia test between healthy highlanders and CMS subjects.

Surprisingly, Peruvian subjects living at 5100 m showed retinal venous constriction during the hypercapnic test independent of the EE status. Results obtained in highlanders contrast with those found in lowlanders, who exhibit a retinal reactivity to CO_2_, evidenced by a 4.2% increase in arteriolar diameter (using the retinal vessel analyser; Dorner et al., 2002) or an increase in arteriolar diameter of 3.3% and in retinal blood flow of 24.9% (using the Canon Laser Blood Flowmeter; Venkataraman et al., 2008). Our results strongly suggest that the vasodilatatory capacity is lost in retinal vessels during chronic exposure to high altitude, as previously suggested by another study (Yang et al., 2019), and that this phenomenon is not limited to highlanders with EE.

Peruvian highlanders living at 5100 m did not show choroidal vasoreactivity to hypercapnia, which is similar to what has been reported in a French lowlander population, using the same LDF device and protocol (Gallice et al., 2017). This appropriate response of ChBF to hypercapnia is in favour of maintained regulation of the orthosympathic system projecting on the choroid (Elam et al., 1981; Morgan et al., 1995), counterbalancing the vasodilatory effect of nitric oxide (Petropoulos et al., 2009; Sato et al., 2003) or prostaglandins.

We should acknowledge some limitations. First, given that females were excluded from the studied population, which was recruited in a mining city mostly employing males, more studies are required to assess female responses to chronic hypoxic exposure (Tremblay, 2023). Second, miners from La Rinconada, in addition to chronic severe hypoxia, are exposed to living and working conditions able to influence their health status (e.g., nutrition, pollution), although this is unlikely to differ between groups with or without EE. Third, we lacked a sea‐level control group of Peruvian subjects, which would allow retinal and choroidal measurements to be obtained in lowlanders with a standard haemoglobin concentration for meaningful comparisons with Peruvian highlanders living at 5100 m with increased haemoglobin concentrations. Fourth, we measured ChBF as a relative value in arbitrary units (which prevents direct comparison between groups), with a measurement resolution of 6%–10% (limiting the capacity of the device to detect tiny changes in ChBF) (Mottet et al., 2018; Tonini et al., 2010). In a previous experiment in lowlanders at high altitude (De Abreu et al., 2025), we were able to detect, with the same device, a significant decrease in ChBF [−13% (−20% to 8%)]. Given that hypercapnia is known to increase the retinal arterial and venous diameters by 19%–25%, we were confident that our method to assess vascular responses was adequate to evaluate CO_2_ vasoreactivity and to support our conclusions. Fifth, the relatively low resolution of fundus images. Owing to technical constraints related to high‐altitude travel to a remote settlement, we had to use portable retinography with a lower resolution (2560 pixels × 1920 pixels) than clinic‐level fundus cameras (up to ∼5000 × ∼5000). Once resized, the image had the minimum resolution recommended for VAMPIRE analysis (MacGillivray et al., 2015). This, and the generally limited quality of the retinal images, affected the accuracy of the vascular measurements and resulted in a need for greater manual intervention. Estimates of vessel diameters have an accuracy of ≤1 pixel, depending on diameter (Lupaşcu et al., 2013). The tortuosity measure used in VAMPIRE minimises the effect of changes in resolution (Lisowska et al., 2014) within the variations expected between state‐of‐the‐art fundus cameras, but the effect on CRAE and CRVE remains to be explored. However, we used the same camera for all subjects, which guaranteed the same rescaling and a consistent analysis.

## 5 CONCLUSION

In conclusion, this ophthalmological experimental study in the highest city in the world showed that highlanders exhibited an abnormal response of veins (vasoconstriction) and an absence of response of arteries to hypercapnia, whereas EE was associated with larger retinal vein diameters. The choroidal blood flow response to hypercapnia was not affected by the presence of EE. These data suggest the presence of retinal vascular dysfunction in highlanders, probably induced by chronic exposure to severe hypobaric hypoxia. Given the similarity between retinal and cerebral vascular regulation, more studies are needed in this population to investigate potential retinal and cerebral vascular abnormalities and their functional consequences.

## AUTHOR CONTRIBUTIONS

Conception and design of the experiment: Aymeric Paillisser, Samuel Verges and Christophe Chiquet. Data acquisition: Aymeric Paillisser, Julien Vincent Brugniaux, Florence Berquet, Jeremy De Abreu, Aymeric Paillisser, Paul Robach, Benoit Champigneulle and Samuel Verges. Data analysis: Aymeric Paillisser, Stephen Hogg, Emanuele Trucco, Martial Geiser, Tom Macgillivray, Aymeric Paillisser, Paul Robach, Christophe Chiquet and Benoit Champigneulle. Interpretation of data: Aymeric Paillisser, Julien Vincent Brugniaux, Stephen Hogg, Emanuele Trucco, Martial Geiser, Tom Macgillivray, Benoit Champigneulle, Aymeric Paillisser, Paul Robach, Samuel Verges and Christophe Chiquet. Drafting the work: Aymeric Paillisser and Christophe Chiquet. Revising the work critically for important intellectual content: Julien Vincent Brugniaux, Florence Berquet, Jeremy De Abreu, Stephen Hogg, Emanuele Trucco, Martial Geiser, Tom Macgillivray, Benoit Champigneulle and Samuel Verges. All authors approved the final version of the manuscript and agree to be accountable for all aspects of the work in ensuring that questions related to the accuracy or integrity of any part of the work are appropriately investigated and resolved. All persons designated as authors qualify for authorship, and all those who qualify for authorship are listed.

## CONFLICT OF INTEREST

The authors declared no potential conflicts of interest with respect to the research, authorship and/or publication of this article.

## GENERATIVE AI STATEMENT

The authors confirm that no artificial intelligence tools, including large language models (LLMs), were used in the drafting or revision of this manuscript. All content was conceived, written and approved solely by the authors.

## Data Availability

Data are available upon request to the corresponding author.
